# Framework for the design and delivery of organized physical activity sessions for children and adolescents: rationale and description of the ‘SAAFE’ teaching principles

**DOI:** 10.1186/s12966-017-0479-x

**Published:** 2017-02-23

**Authors:** David R. Lubans, Chris Lonsdale, Kristen Cohen, Narelle Eather, Mark R. Beauchamp, Philip J. Morgan, Benjamin D. Sylvester, Jordan J. Smith

**Affiliations:** 1 Priority Research Centre for Physical Activity and Nutrition, School of Education, Faculty of Education and Arts, University of Newcastle, Callaghan, 2308 NSW Australia; 2 Institute for Positive Psychology and Education, Faculty of Health Sciences, Australian Catholic University, Strathfield, NSW Australia; 3 School of Kinesiology, Faculty of Education, The University of British Columbia, Vancouver, BC Canada; 4 Faculty of Kinesiology and Physical Education, The University of Toronto, Toronto, ON Canada

**Keywords:** Motivation, Fitness, Enjoyment, Self-determination theory, Physical education, Teaching, Coaching

## Abstract

The economic burden of inactivity is substantial, with conservative estimates suggesting the global cost to health care systems is more than US$50 billion. School-based programs, including physical education and school sport, have been recommended as important components of a multi-sector, multi-system approach to address physical inactivity. Additionally, community sporting clubs and after-school programs (ASPs) offer further opportunities for young people to be physically active outside of school. Despite demonstrating promise, current evidence suggests school-based physical activity programs, community sporting clubs and ASPs are not achieving their full potential. For example, physical activity levels in physical education (PE) and ASP sessions are typically much lower than recommended. For these sessions to have the strongest effects on young people’s physical activity levels and their on-going physical literacy, they need to improve in quality and should be highly active and engaging. This paper presents the Supportive, Active, Autonomous, Fair, Enjoyable (SAAFE) principles, which represent an evidence-based framework designed to guide the planning, delivery and evaluation of organized physical activity sessions in school, community sport and ASPs. In this paper we provide a narrative and integrative review of the conceptual and empirical bases that underpin this framework and highlight implications for knowledge translation and application.

## Background

Regular physical activity provides numerous physical and mental health benefits [1, 2]. However, global prevalence data suggest few children and adolescents accrue enough physical activity required to obtain these benefits [3], which may have both immediate and long-term public health consequences [4–6]. The economic burden of inactivity is substantial, with conservative estimates suggesting the global cost to health care systems in 2013 was US$53.8 billion [7]. In light of the global reach and potential health impacts, physical inactivity has been appropriately described as ‘pandemic’ [8].

School-based programs, including physical education (PE) and school sport have been recommended as important components of a multi-sector, multi-system approach to physical activity promotion [9–11]. Indeed, schools are ideal settings for physical activity promotion, as they have access to youth and often possess the facilities, equipment, and personnel required to deliver PE curricula and other programs [11]. Outside of schools, community sports and after-school programs (ASPs) offer further opportunities for young people to be physically active. In the United States, Canada, Australia and the United Kingdom, between half and two-thirds of school-aged youth participate in organized sports outside of school [12]. The frequency and duration of school- and community-based ASPs varies considerably within and between countries, from an hour once or twice per week to five afternoons per week for 2–3 h at a time [13, 14]. However, in 2014 ASPs were attended by over ten million children in the United States [13]. Each of these settings are important for providing young people with opportunities to experience a routine ‘dose’ of physical activity [15]. However, it is also important to recognize their value for achieving affective, motivational, psychosocial and movement skill outcomes [16]. Such outcomes have obvious short-term benefits, but may also help to develop ‘physical literacy’ and thereby support lifelong physical activity participation [17].

Despite demonstrating promise, evidence suggests schools, community sporting clubs and ASPs are not achieving their full potential. For example, physical activity levels in these settings are typically much lower than recommended [18–20], and a considerable proportion of students leave school without having mastered basic fundamental movement skills [21]. This is likely exacerbated by the fact that many of those charged with delivering PE, sports practice, or ASPs have not received the training needed to confidently deliver active, engaging and educative physical activity experiences [11]. The contribution of youth sports to habitual physical activity may also not be as large as commonly thought [22–24]. In a recent study of Danish primary school students [22], differences in objectively assessed physical activity between sports participants and their non-sporting peers were large for soccer and handball. However, participation in basketball, volleyball and gymnastics contributed little to overall physical activity levels, and students participating in these sports were no more likely to meet physical activity guidelines than non-sporting youth [22].

Increasing physical activity is not the only outcome that could be improved within these settings. Common features of PE teacher practice, such as using controlling language (e.g., terms like ‘must’, ‘should’ or ‘have to’ that convey pressure and/or coerce individuals to act in ways that are inconsistent with their sense of self), or using exercise as punishment, can have immediate and long-term impacts on students’ motivation to be active [25–29]. Similarly, sports participation can be instrumental in the physical, social and emotional development of children and adolescents [30]. Yet, the quality of instruction from sports coaches is highly variable, and not all youngsters have positive experiences with sport [31–34]. Indeed, attrition rates for sports participation are substantial [35], particularly during the teenage years, and ‘lack of enjoyment’ and ‘problems with the coach’ are commonly cited reasons for drop-out [36, 37]. Evidently, there is scope to improve the quality of instruction across each of these organized physical activity settings [23, 38].

At present, knowledge from the fields of education, psychology and public health is fragmented, making it difficult for practitioners (i.e., teachers, coaches and instructors) to know which evidence-based strategies they should be implementing. Moreover, this knowledge is often communicated in a manner intended for a specialist audience, within scholarly publications that are either unknown to practitioners or difficult to access due to the cost of subscriptions. There is a need to consolidate the evidence from these various disciplines into a set of guiding principles, using a practical format and simple recommendations that are ‘sticky’ and easy for practitioners to understand and apply.

**Fig. 1:**
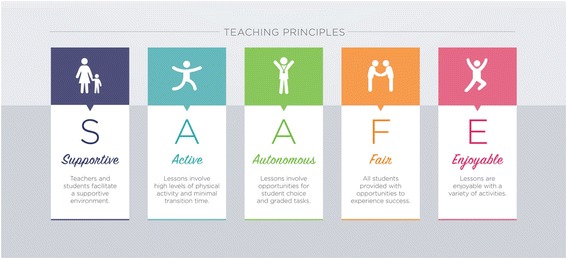
Overview of SAAFE teaching principles

Therefore, the purpose of this paper is to describe the *Supportive, Active, Autonomous, Fair, and Enjoyable* (SAAFE) delivery principles (Fig. 1), an evidence-based framework designed to guide the planning, delivery, and evaluation of organized physical activity sessions in school, after-school, and community sports settings (hereafter referred to as organized physical activity sessions). The SAAFE principles were informed by self-determination theory [39, 40], achievement goal theory [41], competence motivation theory [42, 43] and Epstein’s TARGET framework [Task (design of activities), Authority (distribution of decision-making and student autonomy), Recognition (use of incentives, rewards and feedback), Grouping (formation of students into groups), Evaluation (methods used to assess performance) and Time (appropriateness of workload and lesson pace)] [44, 45]. It should be noted, the SAAFE framework is not the result of a systematic process of evidence synthesis, but rather the product of a large body of empirical evidence, as well as years of collective experience working with teachers, coaches and other physical activity practitioners delivering interventions to young people.

We acknowledge that self-determination theory in particular is being used by researchers and teachers around the world to guide the delivery of organized physical activity sessions [46–48], and guidelines for increasing physical activity in such sessions have emerged in the literature [49, 50]. Indeed, ‘LET US Play’ (Lines, Elimination, Team size, Uninvolved staff or kids, Space, equipment and rules) [50] and ‘SHARP’ (Stretching whilst moving, High repetition of motor skills, Accessibility through differentiation, Reducing sitting and standing, Promoting in class physical activity) [49] are both useful guides for enhancing active learning time within physical activity sessions. However, these guidelines do not address issues related to motivational climates embedded within sessions. We consider the SAAFE principles to be unique as they address the motivational needs of students and the issue of low physical activity levels in organized sessions using a pragmatic set of principles that are easy for teachers to understand and implement.

The SAAFE principles were originally designed to promote a psychologically supportive environment, foster a mastery climate and enhance young people’s autonomous motivation in the Supporting Children’s Outcomes using Rewards, Exercise and Skills (SCORES) primary school physical activity intervention [51, 52]. Our efficacy study showed the SCORES intervention had positive effects on students’ physical activity levels, cardiorespiratory fitness and fundamental movement skills [51]. We are currently testing the effectiveness of a scalable version of the SCORES intervention, called iPLAY (internet-based Professional Learning to help teachers promote Physical activity in Youth) [53]. The SAAFE principles have also since evolved to support the delivery of school-based physical activity interventions targeting adolescents [54–56].

The following section includes a description, rationale and recommended strategies for each of the five SAAFE principles. Practical examples for how practitioners can implement the SAAFE principles are summarized in Table 1. Finally, Table 2 outlines how the SAAFE principles have been applied in three recent school-based physical activity interventions: (i) the SCORES physical activity and movement skills intervention for primary school children [51, 52], (ii) the ATLAS (Active Teen Leaders Avoiding Screen-time) physical activity program for low-active adolescent boys [55, 57, 58], and (iii) the HIIT for Teens (High-Intensity Interval Training for Teens) program, involving the integration of vigorous intensity activity into PE lessons [54, 59].

**Table 1:** SAAFE principles and recommended strategies

Principles	Example strategies
Supportive	• Provide individual skill specific feedback
	• Support feelings of autonomy, competence, and social connection
	• Provide praise on student effort and improvement
	• Acknowledge and reward good sportspersonship
	• Demonstrate empathy toward students who appear frustrated or challenged
Active	• Optimize session structure and activity selection (e.g., small-sided games, multiple games/grids and minimal lines)
	• Avoid elimination activities
	• Include an active warm-up
	• Integrate high-intensity ‘bursts’ of activity within typical games and lesson activities
	• Employ circuits and rotations
	• Complete student registration while students are active
	• Reduce transition time by setting up activities while students are active
	• Minimize teacher talk and instructions
	• Maximize equipment available (e.g., every student with a ball)
Autonomous	• Provide students with opportunities for choice
	• Include free play at the start of sessions
	• Involve students in creation and modification of activities and rules
	• Provide a meaningful rationale for the different activities
	• Minimize controlling language
Fair	• Ensure that students are evenly matched in activities
	• Modify activities to maximize students’ opportunities for success
	• Encourage self-comparison rather than peer-comparison
	• De-emphasize competition (e.g. implement point system that rewards team values and not winning)
	• Regularly change teams/partners (if necessary) to ensure everyone experiences success
Enjoyable	• Design activities with which students can exhibit choice, feel competent, and also interact with others (e.g., group activities)
	• Start and conclude sessions with an enjoyable activity
	• Ensure that sessions involve a variety of tasks/activities
	• Do not use exercise as punishment
	• Use self-selected and motivational music while exercising

**Table 2:** Examples of the SAAFE teaching principles applied in school-based physical activity interventions

Principle	Scores	Atlas	HIIT for Teens
Supportive	Teachers learnt about fundamental movement skills and were instructed to provide students with skill specific feedback to improve students’ motor skill proficiency.	Teachers were instructed to provide students with a rationale for improving their muscular fitness during ATLAS sessions.	‘Trainer of the Day’ certificates were awarded to the student who provided their training partner with the highest quality social support during the HIIT session.
Active	Teachers were encouraged to replace full-sided games (e.g., soccer) with small-sided modified games.	Teachers were provided with circuit cards describing body weight and Gymstick™ (elastic resistance training devices) exercises to ensure that all students could be actively engaged during sessions.	HIIT sessions were embedded into existing PE lessons for 8-weeks. HIIT sessions included 30 s of high intensity activity followed by 30 s of rest (while training partner completed the task).
Autonomous	Students were provided with leadership roles (e.g., running activities, setting up and collecting equipment) in PE, school sport and at lunch-time.	Students were encouraged to complete one HIRT workout (i.e., short duration CrossFit-style fitness challenge) each session and could select the level of difficulty (Easy, Moderate or Hard).	Students completed the HIIT sessions with a partner of their choice and were provided with options regarding exercise selection (e.g., running on the spot or jumping jacks) during sessions.
Fair	Teachers were instructed to monitor and modify lessons (i.e., rules and teams) to ensure that games were not dominated by the most competent students.	Teachers were instructed to monitor partner fitness challenges (e.g., shoulder wrestle activity) to ensure that students were evenly matched.	Students wore heart rate monitors during sessions and were encouraged (by training partners and teachers) to achieve >85% of their heart rate maximum. This objective was considered achievable for all students as success was based on effort not absolute fitness.
Enjoyable	Teachers were instructed to avoid boring and repetitive warm-ups (e.g., running around the field) and replace them with enjoyable starter games.	Sessions provided students with opportunities to enhance their resistance training skill proficiency using a variety of teaching approaches including teacher-led, peer-led, and self-directed pedagogies.	High tempo music was played during HIIT sessions to enhance affect, reduce ratings of perceived exertion, and improve energy efficiency.

*Abbreviations*: *SCORES* Supporting Children’s Outcomes using Rewards, Exercise and Skills, *ATLAS* Active Teen Leaders Avoiding Screen-time, *HIIT* High Intensity Interval Training, *HIRT* high intensity resistance training

### Supportive

Social context is integral to learning and motivation in educational settings [60] and is largely shaped by teachers’ language, behaviors and expectations. From a self-determination theory perspective, teachers can influence their students’ motivation by supporting or thwarting basic psychological needs for: (i) *Autonomy*, the need to experience one’s behavior as self-endorsed or volitional; (ii) *Competence*, the need to effectively interact with one’s environment and achieve positive outcomes; and (iii) *Relatedness*, the need to feel supported and connected with others [39, 40, 61].

The *Supportive* principle recommends that both practitioners and young people facilitate a supportive environment during physical activity sessions. In a supportive environment, practitioners provide a range of safe, challenging and enjoyable learning opportunities that nurture students’ needs, interests, choices, curiosities and preferences; and enable them to experience success [39, 62–66]. Practitioners who are facilitative (rather than controlling) are perceived as being autonomy-supportive by students [66]. These teachers are able to take the perspective of their students, provide a rationale for what they are doing, create meaningful connections, use language that is not strict or controlling, and demonstrate emotional support or involvement (e.g., displaying care, empathy, friendliness, understanding, dedication, and dependability) [60, 66–69].

By contrast, a performance climate promotes the perception that superior performances or winning are the most highly valued outcomes [67, 70–72]. An unsupportive or controlling physical activity environment undermines positive functioning because it elicits feelings of pressure, judgement, and threat among students [63, 66, 73]. In a controlling environment, teachers may be perceived as emotionally closed, and exhibit behaviors that interfere with or bypass students’ inner motives (in an attempt to control what students should think, feel, and do). They may even try to build extrinsic motivation by offering incentives or threatening consequences, using authoritarian language or neglecting students who demonstrate negative affect [66, 74]. Teachers may at times defer to controlling instructional styles as a means of managing ill-discipline or misbehavior. However, prior evidence suggests supportive instructional practices result in students being better adjusted and more engaged in school [75]. Consequently, we believe there is sufficient empirical support to suggest applying the ‘Supportive’ principle is also a useful approach for preventing student misbehavior.

The effective use of feedback in organized physical activities can also greatly impact students’ motivation, engagement, enjoyment and persistence in a task, perceptions of competence, interest in physical activity, motor skill acquisition, and future participation [43, 63, 76–81]. In this context, feedback refers to information given about a performance, and relates to the extent to which the outcome of the performance corresponds to expectations [82]. Providing clear and consistent positive informational and prescriptive feedback to students immediately after a performance (rather than controlling or negative feedback), helps to create a supportive physical activity learning environment [83]. Positive feedback is considered to be most effective when: (i) it is perceived by the learner as honest, (ii) success is attributed to effort and strategy rather than innate ability, (iii) it reinforces improvement and learning rather than social comparison, (iv) is delivered privately rather than publicly (where possible), and (v) the criteria needed to gain positive feedback are specific and achievable, and are made explicit to learners beforehand [79]. Importantly, the amount and nature of feedback should be adjusted to suit the experience and skill level of the performer (i.e., novice learners will typically require more frequent feedback and encouragement) [84]. Moreover, feedback should be used judiciously, as some learners may enjoy the challenge of improving their performances without assistance.

### Active

Our *Active* principle suggests that physical activity sessions should involve high levels of physical activity and minimal transition time. It is important to note that organized physical activity sessions can have direct and indirect effects on young people’s physical activity levels and both should be considered when designing sessions. The direct benefits refer to the ‘dose’ of physical activity provided within sessions, while the indirect benefits relate to additional activity that occurs outside of the sessions resulting from the motivation, knowledge, and skills acquired. The Centers for Disease Control and Prevention (CDC) [85] has previously recommended that students should be engaged in MVPA for at least 50% of PE lesson time. Similarly, the National Institute on Out-of-School Time recommend that ASPs dedicate 30 min (or 20%) of program time to physical activity opportunities, and that at least 50% of this scheduled time be spent in MVPA [86].

Yet, activity levels in PE and other organized sessions are often low. For example, recent systematic reviews of studies examining activity levels in PE have found that students engage in activity for approximately 40% of lesson time in primary [87] and secondary [88] school lessons (ranging from 57.6 to 32.6% when assessed using direct observation and accelerometers). Fewer studies have examined activity levels in ASPs and community sport, but the available evidence suggests that they are not reaching their potential. For example, Beets and colleagues [18] reviewed 25 diverse ASPs in the United States and found that only 16.5% of daily observations satisfied the physical activity target (i.e., at least 4600 steps).

Recently, Lonsdale and colleagues [89] published a systematic review and meta-analysis of interventions aimed at increasing MVPA during PE lessons. Previous interventions have resulted, on average, in a 24% relative increase in the amount of lesson time spent in MVPA. Strategies to increase activity levels in PE can be classified into three broad categories: (i) reducing transition time (e.g., minimizing teacher talk, having more efficient transitions), (ii) maximizing opportunities for activity (e.g., selecting active games, removing elimination), and (iii) fitness infusion (e.g., integrating high-intensity ‘bursts’ of activity within typical games and lesson activities). ‘Fitness infusion’ was found to be the most effective strategy (61% more MVPA time compared with 14% increase associated with other interventions) [89]. However, it stands to reason that implementing all of these strategies concurrently will result in the greatest increase in active learning time.

Activity-promoting instructional strategies have also been implemented within organized sport [90] and ASP settings [91]. For example, Weaver and colleagues [50] designed the ‘LET US Play’ (Lines, Elimination, Team size, Uninvolved staff and children, Space, equipment and rules) principles, which have been used to guide the practice of PE teachers and after-school program staff responsible for delivering games and activities to youth. LET US Play is a useful framework for planning and conducting physical activity sessions, and previous research has shown significant improvements in children’s physical activity in programs when these principles have been applied [92, 93]. Similar instructional practices formed a key part of the HEALTHY school-based intervention [94]. As part of the PE-based component of HEALTHY, teachers were provided with an activity promoting lesson plan, and simple instructional strategies to maximize active time during lessons [95].

### Autonomous

The *Autonomous* principle is focused on the importance of providing students with choice and being offered graded tasks. Many psychological theories highlight the significance of perceived competence and social support for motivation and the development of behavioral intentions [41–43, 96]. However, self-determination theory is noteworthy in emphasizing the critical importance of perceived ‘autonomy’. Self-determination theory posits that autonomy is a fundamental psychological need influencing motivation, behavior, and wellbeing [97]. The theory suggests that in addition to perceived competence and social connection, supporting perceptions of autonomy will promote autonomous forms of motivation, which in turn predict behavioral engagement and persistence. Autonomous motivation refers to a high quality, volitional type of motivation characterized by engaging in behavior that is valued, personally relevant, and enjoyable [39]. Within the physical activity context, previous research has shown that autonomous forms of motivation are more strongly associated with physical activity behavior than controlled forms [98]. Controlled motivation refers to engaging in behavior due to internal or external pressures (e.g., lunch-time detention) [39].

Within the SAAFE framework, the *Autonomous* principle focuses largely on the importance of choice, alongside the elements of autonomy-supportive teaching (e.g., providing a rationale and taking the perspective of the student) aligning with the *Supportive* principle previously described. Consistent evidence across many life contexts indicates that people who perceive they can make meaningful choices are likely to be intrinsically motivated, meaning that they are more likely to find activities enjoyable and interesting [99]. Within physical activity contexts, in particular, students who perceive that they have greater choice also are more intrinsically motivated and ascribe greater value to physical activity compared with students who feel their autonomy is undermined [100]. Experimental evidence shows that providing students with the opportunity to select their activities from a range of options provided by the teacher increases their total physical activity during PE lessons [101, 102]. Furthermore, providing students with brief periods of complete free choice increases their MVPA compared with a lesson led by the teacher [101, 102]. Free play for children is an important end in itself, but also promotes a variety of positive social, emotional and cognitive outcomes. Promoting free play is perhaps even more valuable in an era of increasing urbanization and fearful parenting practices [103, 104].

The number of ways in which choice can be incorporated into physical activity sessions are likely only limited by the teachers’ imagination. Table 1 outlines ways in which teachers have been encouraged to provide choice in our recent interventions. Along with these possibilities, we also suggest that teachers carefully consider the way in which they provide opportunities for students to make choices and decisions. For example, we recommend that teachers avoid providing too many options, as students may find this burdensome and de-motivating [105]. Based on meta-analytic evidence [99], two to four opportunities for choice within a session is ideal. When offering opportunities for complete free choice, we suggest somewhere between 5 and 10 min at the start of a session is a sufficient amount of time for students to play without direct instruction, and this duration enables teachers time to set up and structure activities that are linked to the core objectives of the session.

The ‘types’ of choices that are offered to students should be considered carefully. Allowing student captains to select team members during PE could be viewed as supporting choice. However, the experience of being selected last can be traumatizing for students, and these experiences may have prolonged adverse impacts on physical activity participation [106]. Consequently, a common-sense approach, that also considers the potential harms of enabling certain choices, should be applied when planning for the provision of choice. Importantly, practitioners should provide both ‘option choice’ (e.g., selection of activity) and ‘action choice’ (e.g., control of the pace of task progression). Although option choice might be easier to plan and deliver, previous research suggests action choice is more effective for enhancing intrinsic motivation [107]. In light of this, it is important that instructors not rely on option choice alone as a means of providing autonomy support.

### Fair

Our *Fair* principle is concerned with providing all students with opportunities to experience success in the physical domain. It is important to note that success (mastery) and having fun (enjoyment) are not synonymous constructs/outcomes (although both are inter-related), and that both are important targets for promoting physical activity engagement. Consistent with the idea of a mastery climate, we view success to be synonymous with personal improvement and not satisfaction of an absolute level of physical performance. PE classes, youth sporting teams, and ASP groups will often include individuals across the continuum of physical ability. Despite this, the manner in which teachers plan and deliver physical activities can have an impact on perceptions of fairness among participating youth. Perceptions of fairness have been shown to influence motivation and affective learning [108], enjoyment [109] and intentions to continue participating in sports [109, 110]. Consequently, it is critical that teachers consider how their practices either support or undermine these perceptions.

Competition is a core component of many physical activities, and introducing competition can make activities motivating and engaging (assuming that success appears achievable for all). Although competing in team games requires youth to demonstrate a number of desirable behaviors (e.g., cooperation, communication, effort etc.), students typically equate competition purely with winning and losing [111]. It is therefore important for teachers to use competition judiciously, and to consider whether their instructions and feedback are promoting a performance climate (i.e., a narrow ‘win or lose’ view of competition is reinforced) or mastery climate (e.g., effort and personal improvement are valued over winning). Of note, there is considerable developmental variability among young people of the same chronological age [112], which has important implications for mastery experiences during competitive activities, and subsequently on the development of physical self-concept. Evidence from elite sport has demonstrated the ‘relative age effect’ is a worldwide phenomenon that exists in many competitive sports, whereby children born early in the competition year have a competitive advantage over their younger peers [113, 114]. To promote fairness, teachers are encouraged to consider maturational differences, particularly for youth near the pubertal period where such differences become increasingly pronounced. Although maturational differences cannot be prevented, practitioners can be cognizant of the influence of differences in size, speed or strength when organizing competitive tasks, providing feedback to youth, or praising successful performance.

Equity is important in coeducational physical activity contexts, as the physical and experiential advantage that boys often possess can disadvantage their female peers [115]. Indeed, the dominance of boys in activities during coeducational PE has been identified as a key barrier to female participation and enjoyment [116]. Such differences may be one factor explaining why girls typically enjoy PE less and experience greater declines in PE enjoyment over time, compared with boys [117]. There is evidence to suggest that reinforcing a mastery climate in PE is a useful way for teachers to enhance students’ experiences and perceptions of equity, regardless of biological sex [118]. We recommend that teachers deliver a diverse range of activities that appeal to all students, regardless of their ability levels and motivation. Of note, fear of negative social evaluation and teasing from boys commonly discourages girls from participating in coeducational PE lessons [116]. Moreover, single sex groups have been shown to result in greater participation among girls, and more frequent verbal feedback to girls from the teacher [119]. Therefore, the separation of classes into single-sex groups and/or allowing students to select the level of competition in game-based activities (i.e., students can choose to participate in a recreational or competitive game), might be useful for supporting girls’ participation.

Students with physical and intellectual disabilities are often disadvantaged in physical activity contexts. Mobility, vision, and hearing impairments are obvious impediments to the successful performance of physical activities. In addition, motor coordination deficits are a hallmark feature of intellectual and developmental disabilities such as autism spectrum disorder and dyspraxia [120]. Of concern, the physical activity experiences of many students with a disability include outright exclusion, tokenistic inclusion (e.g., role as line judge or score keeper), and unfair performance expectations [121]. To promote the equitable treatment of all youth in physical activity sessions, it is critical that teachers adapt activities to suit their various needs. We recommend that teachers plan for and deliver adapted physical activities that enable all students to demonstrate success and progress, regardless of their level of ability. To emphasize, ‘success’ in this context refers to striving for and experiencing personal improvement, regardless of the absolute level of performance, as noted previously. Modifications could include changes to the distance from or size of a target, the use of different equipment (e.g., a larger bat or ball) in drills or games, and changes to game rules that level the playing field for all students (e.g., playing blindfolded games such as ‘goalball’), or at least support participation of students with disabilities (e.g., passive defense rule for student with a mobility impairment playing basketball).

It is also recognized that the level of expertise required to adapt lessons for students with disabilities is challenging for many teachers. However, in some countries (e.g., Australia), students with special educational needs are integrated into mainstream classes. Therefore, professional learning, and/or additional trained support staff may be needed to facilitate adapted lesson delivery. Outside of these training opportunities, teachers and instructors can actively consult with learners and their parents/carers to determine appropriate and feasible modifications that can be made during lessons, and to demonstrate to youth with disabilities that they are not being forgotten in these physical activity contexts.

### Enjoyable

The *Enjoyable* principle directly aligns with prominent theories of motivation, which purport that people tend to persist with activities they find intrinsically motivating [122]. When people pursue physical activities (or indeed any other activity) for the inherent joy and pleasure, they are said to be intrinsically motivated which, in turn, tends to result in greater adherence to and pursuit of those behaviors [123, 124]. Indeed, enjoyment has been a consistently reported mediator/mechanism of the effects of efficacious physical activity interventions among youth [25, 125].

In terms of the (social) conditions that promote physical activity enjoyment, research from different theoretical perspectives point to a consistent cluster of strategies that those concerned with physical activity promotion can harness. From the perspective of self-determination theory [122], and as highlighted under the *Supportive* principle, when children and adolescents feel autonomous, socially connected to others, and competent they are more likely to enjoy the activity [100]. In the context of youth sport [126] and PE [46, 127], when children and adolescents are provided with the opportunity to exercise some choice, they tend to report greater engagement, greater future intentions for physical activity, and greater persistence in the activity [128]. Similarly, when youth feel socially connected to their coach or other children in a class or sports team [129] they tend to have greater satisfaction and positive emotions. Finally, when social agents such as coaches and teachers structure the physical activity environment to maximize feelings of competence and personal mastery, children are more likely to enjoy the activity, maintain interest in involvement, and commitment to the activity [43].

In addition to those strategies that enable youth to feel autonomous, competent, and socially connected to those within their social milieu, recent research has sought to examine the efficacy of other strategies and psychological experiences that might translate into people enjoying physical activity to a greater extent [130]. For example, when children are provided with a greater variety of exercise equipment (compared to less variety) in a single bout of exercise, they report greater enjoyment of that exercise, and participate in more exercise behavior [131, 132]. Furthermore, using an experimental design, Sylvester and colleagues found that when a 6-week exercise program was structured to involve greater variety (otherwise known as variety support), participants subsequently experienced greater adherence [133], as well as improved psychological well-being (greater positive affect and subjective vitality and lower negative affect) [134] than those participants randomized to a program that was devoid of such variety.

In addition to lab-based studies, recent examination of the Pokemon Go phenomenon (the most downloaded game in US history) has pointed to the provision of variety within its platform that fosters such high usage of this exergame [135]. Other (non-experimental) work has similarly examined the role of novelty in PE settings, and found that novelty is associated with intrinsic motivation [136]. Finally, an adjunct strategy that appears to demonstrate considerable appeal in supporting physical activity participation is the use of music. When utilized independently (i.e., without physical activity), and as is evident from the millions of people that report enjoying it, music has consistently been found to foster improvements in affective states [137]. When coupled with repetitive and aerobic (endurance-type) physical activities, the use of self-selected and motivational music has been found to result in improvements in affective responses [138]. These effects are particularly pronounced when used with self-paced exercise. We recommend the use of music, where appropriate (e.g., during fitness circuits), to enhance engagement but also caution against this strategy if the distracting effects of music might undermine the learning objectives. As a final note, when asked what they want from a physical activity intervention, youth emphasize the critical importance of ‘fun’ [139, 140]. These findings point to a cautionary note against using physical activity as a form of punishment. Associating physical activity with punishment is unlikely to promote a sense of fun, and may undermine the feeling that physical activity is an avenue for pursuing enjoyment.

## Conclusions

As identified in recent reviews [141], there is a clear need for the effective dissemination of evidence-based physical activity strategies. Recommended strategies include: creating partnerships with educational authorities to deliver professional learning workshops for teachers, presentations at practitioner conferences, increased focus on intervention dissemination and scaling-up research, and imbedding evidence-based pedagogical practices in pre-service teacher education courses. The SAAFE principles and practical strategies have been designed to enable practitioners to deliver engaging physical activity sessions to youth, in a manner that maximizes physical activity participation and promotes physical literacy by enhancing affective, cognitive, motivational, and movement skill outcomes. Teachers, coaches, facilitators and instructors are encouraged to: (i) be *Supportive* in their teaching, (ii) maximize students’ opportunities to be physically *Active*, (iii) create an *Autonomous* learning environment by including elements of choice and providing a rationale for activities, (iv) design and deliver lesson experiences that are *Fair* by allowing all students to experience success regardless of their physical abilities, and (v) provide an *Enjoyable* experience by focusing on fun and variety.
